# The Effect of Monthly Anti-CD25^+^ Treatment with Basiliximab on the Progression of Chronic Renal Dysfunction after Lung Transplantation

**Published:** 2020

**Authors:** D. J. Ross, J. Belperio, C. Natori, A. Ardehali

**Affiliations:** 1 *Division of Pulmonary/Critical Care Medicine/Clinical Allergy & Immunology; David Geffen-UCLA School of Medicine, Los Angeles, CA, USA*; 2 *Department of Nursing/Transplant Administration/Ronald Reagan-UCLA Medical Center, Los Angeles, CA, USA*; 3 *Division of Cardiothoracic Surgery; David Geffen-UCLA School of Medicine, Los Angeles, CA, USA*

**Keywords:** Renal Insufficiency, Chronic, Calcineurin Inhibitors, lung transplantation, Basiliximab, Immunosuppression

## Abstract

**Background:**

Chronic renal dysfunction (CRD), as predominantly related to calcineurin-inhibitor (CNI) nephrotoxicity, is associated with increased morbidity and mortality after lung transplantation (LTx). Basiliximab (BSX), a recombinant chimeric monoclonal antibody against CD25^+^ on activated T-lymphocytes, although often employed as an “induction immunosuppression” after solid organ transplantation, may further allow for reduction in CNI exposure with monthly administration and amelioration of CRD.

**Objective:**

To determine the effect of monthly anti-CD25^+^ treatment with basiliximab on the progression of chronic renal dysfunction after lung transplantation.

**Methods:**

Post-LTx recipients with stages IIIB-V CRD were treated with monthly intravenous infusion of BSX 20 mg. They were analyzed for creatinine clearance at 1, 3, 6, and 12 months; rate of the change in the clearance (the slope of the regression line) and FEV_1_/month; de novo HLA class I or II DSA; and infectious events (IE). Tacrolimus (TAC) trough levels were concurrently targeted at 2–4 ng/mL during BSX therapy. The criteria for BSX discontinuation included acute lung allograft rejection, acute respiratory infection, and progression to end-stage renal disease (ESRD).

**Results:**

9 LTx recipients were treated with BSX for ≥6 months. The median time past after their LTx was 1853 (range: 75–7212) days; the mean±SD age was 64.3±11.3 years; the male:female ratio was 7:2. The baseline mean±SD creatinine clearance 1–3 months prior to BSX initiation was 22.8±5.14 mL/min/1.73 m^2^ (CI: 3.95) consistent with CRD stages—IIIB (2), IV (6), and V (1). Prior to BSX treatment, all 9 patients had established CLAD—obstructive-phenotype (BOS, n=4) and restrictive-phenotype (RAS, n=5). During the course of BSX treatment, the aggregate creatinine clearance mean slope increased by a mean±SD of 0.747±0.467 mL/min/1.72 m^2^/month (CI: 0.359), consistent with “stabilization” of renal function in 7 patients; deterioration occurred in 2 with transition to chronic hemodialysis. Spirometric stability in lung allograft function was observed in 5 patients with a mean±SD aggregate FEV_1_ slope of -1.49±1.08 mL/month (CI: 2.50). 3 deaths occurred due to the following conditions during BSX treatment—HFpEF/Sepsis + CLAD/Parainfluenza type 2 bronchiolitis + CLAD. 2 recipients developed “weak MFI” HLA class II DSA; no HLA class I DSA was detected during the treatment.

**Conclusion:**

Renal sparing therapy with monthly BSX infusion with concurrent reduction in CNI exposure (TAC = 2–4 ng/mL) for stages IIIB-V CRD was associated with stability in creatinine clearance in 78% of patients over a treatment course of 6–12 months. Pre-existing CLAD afflicting all patients and inherent variability in progression of chronic rejection, limits our assessment of BSX efficacy in this context. We detected an infrequent de novo HLA class II DSA during BSX therapy.

## INTRODUCTION

Extra-pulmonary complications of lung transplant (LTx) and chronic immunosuppression are myriad, including the development of chronic renal dysfunction (CRD) [1-4]. Calcineurin-inhibitor (CNI) therapy is the predominant etiology, however, additional contributions have been elucidated, such as other nephrotoxic medications, hypertension, diabetes mellitus, and polyoma BK virus-related nephropathy (BKN) [1, 4]. CRD is common after solid organ transplantation and may afflict 30%–50% of non-renal transplant recipients while associated with increased morbidity and mortality [3]. Strategies to ameliorate CRD have targeted a reduction in CNI exposure and potential avoidance or replacement by a less-nephrotoxic immunosuppressant regimen such as incorporating mammalian target of rapamycin (mTOR) inhibitor [4, 5]. Decisions regarding the alteration in CNI-based regimen may further be influenced by clinical concern for precipitating acute or chronic lung allograft dysfunction/rejection (CLAD) and/or development of de novo donor-specific allo-antibodies (DSA). 

Basiliximab (BSX), a recombinant chimeric murine-human IgG1 monoclonal antibody against CD25^+^ antigen on activated T-lymphocytes, is frequently implemented as “induction immunosuppression” after solid organ transplantation while permitting the optimization of therapeutic CNI targeted blood levels [6]. BSX effectuates complete saturation of IL-2Rα with a BSX concentration >0.2 mg/mL that is sustained for approximately 4–6 weeks without any associated myelosuppression and rare anti-idiopathic antibodies [7-9]. We have observed frequent and significant co-morbidity of CNI associated with CLAD, thereby contributing to a clinical dilemma—whether to reduce CNI exposure with potential risk of contributing to further decline in allograft physiologic function. We hypothesized a potential utility of BSX, during an era of reduced CNI exposure with tacrolimus (TAC), may be associated with improvement or stabilization in renal dysfunction but without compromising lung allograft function or contributing to development of de novo HLA DSA. 

## MATERIALS AND METHODS

The Inclusion Criteria

POST-LTx patients considered for monthly BSX therapy at our center included those with (1) progressive stages IIIB-V chronic renal disease; (2) clinically suspected CNI-induced chronic nephrotoxicity; (3) absence of BK viremia; (4) prior determined lack of renal functional improvement associated with reduction in target TAC trough level to 5–7 ng/mL; (5) absence of acute respiratory infection; (6) clinical availability for monthly outpatient intravenous administration of BSX; and (7) optimized on either angiotensin converting enzyme-inhibitor or angiotensin II receptor-inhibitor therapies.

Protocol

Patients were initiated on monthly administration of BSX 20 mg iv, as an outpatient. The target TAC trough levels were then reduced to 2–4 ng/mL; doses of mycophenolate mofetil were maintained per routine clinical protocol and adjustment for total WBC ≥3000 was made. Prednisone was maintained as maintenance dose of approximately 0.1 mg/kg/day. Antibiotic prophylaxis for Pneumocystis jirovecci was maintained per routine clinical protocol with either sulfamethoxazole/trimethoprim or atovaquone; anti-fungal suppression as clinically indicated based on prior respiratory mycosis isolation; CMV prophylaxis maintained per routine clinical protocol—CMV donor: (+)/Recipient: (+): valganciclovir x 6–12 months/CMV donor: (+)/Recipient: (–) serologic status: indefinite valganciclovir prophylaxis/CMV Donor: (–)/Recipient: (–) status: acyclovir prophylaxis. All patients received CLAD prophylaxis with routine azithromycin 250 mg three times a week. Laboratory monitoring was maintained per routine clinical protocol with chemistry, CBC, TAC trough level, CMV DNA quantitative PCR approximately every 4–6 weeks, BK virus serum quantitative PCR and single-antigen flowcytometry HLA class I and II allo-antibodies (Luminex^®^) every 3–4 months. 

Primary End-point: Stability or Improvement in Renal Function

Assessment of creatinine clearance as calculated by the Cockcroft-Gault formula was performed at 1, 0, 3, 6, 9, and 12 months relative of the initiation of monthly administration of BSX. The individual patient slopes were calculated for the clearance against the time stratified by the baseline CRD category according to the NKF/DOQI guidelines [10-12]. 

Exploratory Secondary End-points: Stability of Lung Allograft Physiologic (Spirometric) Function

Assessments of spirometric function were performed per routine Post-LTx protocol and clinical indications per American Thoracic Society (ATS) standards. FEV_1_ vs. time trends and slopes were calculated [13]. Patients were categorized by the type and severity of their CLAD, in accordance with guidelines developed by the International Society of Heart and Lung Transplantation as “bronchiolitis obliterans syndrome” (BOS) or “restrictive allograft syndrome” (RAS) [14, 15]. 

Discontinuation Criteria for BSX

(1) Acute lung allograft rejection (AR); (2) acute respiratory infection; (3) progression to ESRD/CKD stage V with initiation of hemodialysis (HD). 

## RESULTS

Cohort Characteristics

There were patients treated with monthly BSX (Table 1). The median time past from LTx was 1853 (range: 775–7212) days. The baseline mean±SD creatinine clearance was 22.8+5.14 (CI: 3.95) mL/min/1.73 m^2^. The baseline CRD classification upon initiation of BSX treatment was stage IIIB (22%), stage IV (67%), and stage V (11%). All patients had prior evidence for CLAD of varying severity—four with BOS and five with RAS. 

**Table 1:** Patients’ demographics

Patient	Age/Sex	Native Disease	LTx/Days post-	CLAD	CRD Grade
1	72/M	UIP	SLT/1853	CLAD/BOS 2	4
2	56/M	CF	BLT/7212	CLAD/BOS 3	4
3	75/M	HP	SLT/1262	CLAD/RAS 2	3B
4	68/F	CPFE	SLT/2104	CLAD/BOS 1	4
5	72/M	UIP	SLT/1710	CLAD/RAS 1	5
6	38/M	GVHD	BLT/3660	CLAD/RAS 2	4
7	68/M	COPD	SLT/775	CLAD/RAS 2	4
8	63/M	UIP	BLT/1952	CLAD/RAS 3	4
9	67/M	HP	SLT/808	CLAD/BOS 2	3B

UIP: Usual interstitial pneumonitis; COPD: Chronic obstructive lung disease; CF: Cystic fibrosis; HP: Chronic hypersensitivity pneumonitis; CPFE: Combined pulmonary fibrosis + emphysema; GVHD: Chronic graft vs. host disease; LT: Single lung transplant; BLT: Bilateral lung transplant; CLAD: Chronic lung allograft dysfunction; BOS: Bronchiolitis obliterans syndrome; RAS: Restrictive allograft syndrome

BSX Effects on Allograft Function Post-LTx

**Figure 1:**
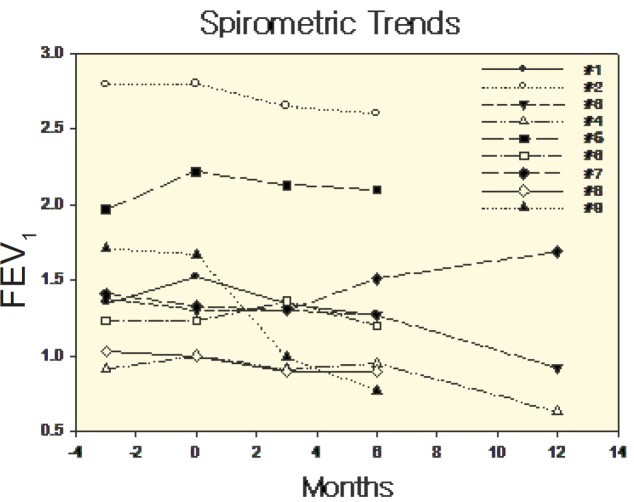
FEV_1_ temporal trends for individual patients

**Figure 2:**
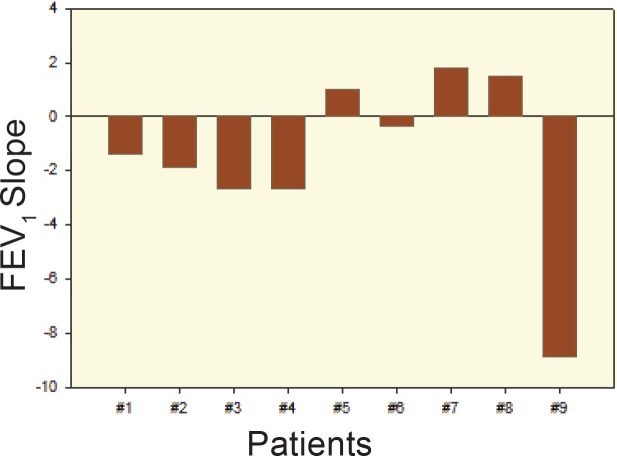
Individual FEV_1_ slope *vs*. time during BSX therapy

The individual patient spirometric trends for FEV_1_ *vs.* time both before and during monthly BSX treatment (t=0) are shown in Figure 1; individual slopes on BSX treatment are presented in Figure 2. The mean±SD slope for FEV_1_ vs. time was 1.49±1.08 mL/month (CI: 2.50) during BSX therapy. Stability in FEV_1_ values was observed in five patients. During immune surveillance with single antigen flowcytometry for HLA-associated DSA, at the 6-month epochs, only two of the nine Patients developed de novo HLA class II DSA, which were determined as “weak” allo-antibodies by Median Fluorescence Index (MFI); no HLA class I DSA was detected. 

**Figure 3:**
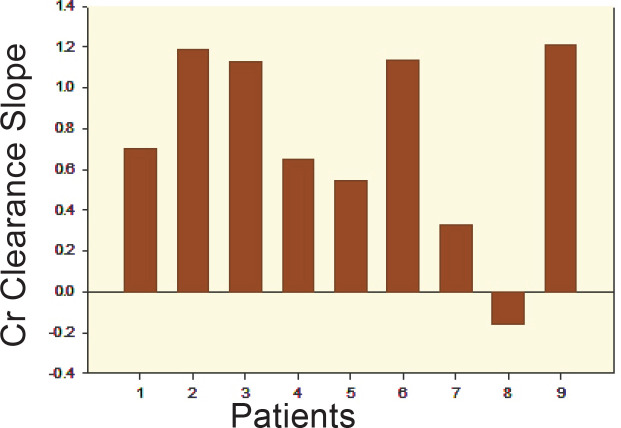
Individual creatinine clearance slope *vs*. time during BSX therapy

BSX Preservation of Renal Function and Clinical Course

The effects of BSX and progression of CRD are presented in Figure 3 whereupon for the cohort, the aggregate creatinine clearance increased in eight patients as manifested by a positive mean±SD slope of 0.747±0.467 mL/min/1.73 m^2^/month (CI: 0.359). Individual patient vignettes are outlined in Table 2. Deaths occurred in three of the nine patients; no mortality was determined as related specifically to the BSX therapy—one death was attributed to progressive CLAD/BOS with pneumonia approximately 11.5 months on BSX therapy; one related to exacerbation of CLAD and potential sepsis at 9 months; and another due to parainfluenza type II bronchiolitis with progressive CLAD/BOS at 8 months on BSX. In summary, monthly BSX therapy was associated with stability in spirometric parameters in five of the nine patients and stability in renal function in seven—two patients progressed to ESRD and transitioned to chronic hemodialysis. 

**Table 2:** Individual patient vignettes

1- Improved creatinine clearance/stable spirometry/BOS 2/C/B HFpEF/(–) DSA
2- Slow decrement creatinine clearance/stable spirometry/BOS 3/(–) DSA
3- Improved creatinine clearance/progressive R-CLAD → D/C BSX (12 months)/(–) DSA
4- Stable creatinine clearance (12 months)/progressive BOS 1 → 2/C/B HFpEF/Pneumonia (Deceased)
5- Improved creatinine clearance/stable R-CLAD (7 months)/(+)*de novo *HLA class II DSA/C/B viral PNA → D/C BSX after 7 months → IVIG + rituximab
6- Improved creatinine clearance/stable R-CLAD/(–) DSA
7- Stable creatinine clearance (16 months) → ESRD + HD/C/B recurrent CHF + HFpEF + possible AR → D/C BSX → rabbit ATG with stable spirometry (BOS 0)/(–) DSA
8- Stable creatinine clearance (4 months) C/B AKI + CKD → HD/stable R-CLAD → C/B sepsis + acute + chronic respiratory failure at 6 months (Deceased)/(–) DSA
9- Improved creatinine clearance/C/B Parainfluenza-II PNA + BOS 3 → (Deceased) after 8 months/weak (+) *de novo *HLA class II DSA

ATG: Anti-thymocyte globulin; HFpEF: Heart failure with preserved ejection fraction; DSA: Donor-specific HLA antibodies; BOS: Bronchiolitis obliterans syndrome; RAS: Restrictive allograft syndrome; AR: Acute rejection; AKI: Acute kidney injury; ESRD: End-stage renal disease

## DISCUSSION

In our preliminary single-center experience utilizing a strategy of reduction in CNI exposure and monthly intravenous administration of anti-CD25^+^ therapy with BSX for renal sparing, we found a reduction in the progression of CRD as demonstrated by a positive slope of creatinine clearance over time and stability in seven of nine studied patients. An increased prevalence of infectious complications post-LTx are well described, in particular, when pre-existent CLAD afflicts the allograft. Therefore, it would be difficult to ascertain from our experience, the contribution of BSX to the observed respiratory infections [16-19]. In a placebo-controlled randomized trial on 340 renal transplant recipients who were treated with either BSX induction vs. the standard triple-drug immunosuppression, no differences were observed in overall infectious complications or CMV infections [20]. 

Strategies typically implemented in an attempt to ameliorate CRD post-LTx may include (1) reduction in CNI target levels; (2) addition or substitution of rapamycin, an mTOR-inhibitor; (3) discontinuation of proton pump-inhibitors (PPI); (4) optimization of hypertension and diabetes mellitus; and (5) avoidance of nephrotoxic medications, when clinically feasible. In our series, all patients had pre-existing evidence for CLAD (i.e., chronic rejection), thereby raising concern of potential adverse effects of a reduction in targeted CNI levels on allograft function. BSX treatment was associated with stability in FEV_1_ in five of nine patients with pre-existing CLAD. In our series, the effect of BSX on the natural history of CLAD could not be specifically determined. However, one would typically anticipate a progressive deterioration in allograft function. In a small clinical series (n=15) of older LTx candidates with pre-existent CRD who had received BSX induction vs. a historical control group, there was a trend for reduction in both acute cellular rejection and CLAD without increase in either infectious complications or malignancy [21]. We would therefore propose monthly BSX administration and concurrent reduction in CNI exposure, as an additional clinical option while assessing the risks vs. benefits of CNI reduction with maintenance of allograft functional integrity. 

Limitation to our preliminary report, includes the small study sample size and the inherent issues associated with an uncontrolled clinical series. A matched placebo-control study, although preferred, would be a challenge to envision in the context of the severity in patient co-morbidities with CKD and CLAD. Furthermore, we debated an ethical concerns and the appropriateness of a protocol which stipulates reduction in TAC trough levels for a control group who are afflicted by pre-existent chronic rejection but without introducing an alternative adjunctive immunosuppressant therapy. We would therefore propose future consideration of a multi-center, controlled, clinical trial with a reduced TAC trough level of 2–4 ng/mL + mycophenolate + prednisone + monthly BSX infusion vs. reduced TAC trough level of 2–4 ng/mL + mycophenolate + prednisone + rapamycin (an mTOR inhibitor).

## References

[1] Hogerle BA, Kohli N, Habibi-Parker K (2016). Challenging immunosuppression treatment in lung transplant recipients with kidney failure. Transpl Immunol.

[2] Osho AA, Hirji SA, Castleberry AW (2017). Long-term survival following kidney transplantation in previous lung transplant recipients-An analysis of the unos registry. Clin Transplant.

[3] Shashaty MGS, Forker CM, Miano TA (2019). The association of post-lung transplant acute kidney injury with mortality is independent of primary graft dysfunction: A cohort study. Clin Transplant.

[4] Sole A, Zurbano F, Borro JM (2015). Prevalence and Diagnosis of Chronic Kidney Disease in Maintenance Lung Transplant Patients: ICEBERG Study. Transplant Proc.

[5] Stephany BR, Boumitri M, Budev M (2009). Absence of proteinuria predicts improvement in renal function after conversion to sirolimus-based immunosuppressive regimens in lung transplant survivors with chronic kidney disease. J Heart Lung Transplant.

[6] Chapman TM, Keating GM (2003). Basiliximab: a review of its use as induction therapy in renal transplantation. Drugs.

[7] Kapic E, Becic F, Kusturica J (2004). Basiliximab, mechanism of action and pharmacological properties. Med Arh.

[8] McKeage K, McCormack PL (2010). Basiliximab: a review of its use as induction therapy in renal transplantation. BioDrugs.

[9] Kovarik JM, Kahan BD, Rajagopalan PR (1999). Population pharmacokinetics and exposure-response relationships for basiliximab in kidney transplantation The US Simulect Renal Transplant Study Group. Transplantation.

[10] National Kidney F (2002). K/DOQI clinical practice guidelines for chronic kidney disease: evaluation, classification, and stratification. Am J Kidney Dis.

[11] Levey AS, de Jong PE, Coresh J (2011). The definition, classification, and prognosis of chronic kidney disease: a KDIGO Controversies Conference report. Kidney Int.

[12] Levey AS, Tangri N, Stevens LA (2011). Classification of chronic kidney disease: a step forward. Ann Intern Med.

[13] Gardner RM, Hankinson JL (1988). Standardization of spirometry--1987 ATS update (American Thoracic Society). J Occup Med.

[14] Glanville AR, Verleden GM, Todd JL (2019). Chronic lung allograft dysfunction: Definition and update of restrictive allograft syndrome-A consensus report from the Pulmonary Council of the ISHLT. J Heart Lung Transplant.

[15] Meyer KC, Raghu G, Verleden GM (2014). An international ISHLT/ATS/ERS clinical practice guideline: diagnosis and management of bronchiolitis obliterans syndrome. Eur Respir J.

[16] Chan CC, Abi-Saleh WJ, Arroliga AC (1996). Diagnostic yield and therapeutic impact of flexible bronchoscopy in lung transplant recipients. J Heart Lung Transplant.

[17] Chan KM, Allen SA (2002). Infectious pulmonary complications in lung transplant recipients. Semin Respir Infect.

[18] Palmer SM Jr, Henshaw NG, Howell DN (1998). Community respiratory viral infection in adult lung transplant recipients. Chest.

[19] Sims KD, Blumberg EA (2011). Common infections in the lung transplant recipient. Clin Chest Med.

[20] Ponticelli C, Yussim A, Cambi V (2001). A randomized, double-blind trial of basiliximab immunoprophylaxis plus triple therapy in kidney transplant recipients. Transplantation.

[21] Borro JM, De la Torre M, Miguelez C (2005). Comparative study of basiliximab treatment in lung transplantation. Transplant Proc.

